# Advances in Radionuclides and Radiolabelled Peptides for Cancer Therapeutics

**DOI:** 10.3390/pharmaceutics15030971

**Published:** 2023-03-17

**Authors:** Kushal Chakraborty, Jagannath Mondal, Jeong Man An, Jooho Park, Yong-Kyu Lee

**Affiliations:** 1 Department of IT and Energy Convergence (BK21 FOUR), Korea National University of Transportation, Chungju 27469, Republic of Korea; 2 Department of Green Bio Engineering, Graduate School, Korea National University of Transportation, Chungju 27469, Republic of Korea; 3 4D Convergence Technology Institute, Korea National University of Transportation, Jeungpyeong 27909, Republic of Korea; 4 Department of Bioengineering, College of Engineering, Hanyang University, Seoul 04763, Republic of Korea; 5 Department of Applied Life Science, Graduate School, BK21 Program, Konkuk University, Chungju 27478, Republic of Korea; 6 Research Institute for Biomedical & Health Science, Konkuk University, Chungju 27478, Republic of Korea

**Keywords:** drug delivery, theragnostic, radionuclide, peptide conjugates, anticancer therapy, clinical application

## Abstract

Radiopharmaceutical therapy, which can detect and treat tumours simultaneously, was introduced more than 80 years ago, and it has changed medical strategies with respect to cancer. Many radioactive radionuclides have been developed, and functional, molecularly modified radiolabelled peptides have been used to produce biomolecules and therapeutics that are vastly utilised in the field of radio medicine. Since the 1990s, they have smoothly transitioned into clinical application, and as of today, a wide variety of radiolabelled radionuclide derivatives have been examined and evaluated in various studies. Advanced technologies, such as conjugation of functional peptides or incorporation of radionuclides into chelating ligands, have been developed for advanced radiopharmaceutical cancer therapy. New radiolabelled conjugates for targeted radiotherapy have been designed to deliver radiation directly to cancer cells with improved specificity and minimal damage to the surrounding normal tissue. The development of new theragnostic radionuclides, which can be used for both imaging and therapy purposes, allows for more precise targeting and monitoring of the treatment response. The increased use of peptide receptor radionuclide therapy (PRRT) is also important in the targeting of specific receptors which are overexpressed in cancer cells. In this review, we provide insights into the development of radionuclides and functional radiolabelled peptides, give a brief background, and describe their transition into clinical application.

## 1. Introduction

Because cancer is one of the diseases with the highest mortality rates, the battle against cancer has attracted the attention of many scientists. Researchers have investigated a wide variety of approaches to treat malignant tumours, including photothermal, photodynamic, sonodynamic, and immune therapy [1,2,3,4,5,6,7,8,9,10,11]. Despite the significant developments made in the fight against cancer in recent years, no single therapy has been successful in treating the disease entirely. Better patient selection, prediction of treatment response and tissue toxicity, and response evaluation are all possible through theragnostics, since diagnostic and therapeutic methods relating to the same precise molecular targets can be coupled. Although the term theragnostics was originally put forth back in 2002, the notion underlying theragnostics has been used and explored over the years [12]. Nuclear theragnostics involves the use of radioactive compounds for imaging biological phenomena by detecting the expression of disease-related targets such as cell surface receptors or membrane transporters, followed by the application of agents designed to deliver ionising radiation to the tissues that express these targets. For the purpose of detection, a positron-emitting nuclide is injected intravenously, and the number of positrons emitted in the body is monitored using a detection camera. Images of nuclide distribution provide insight into the illness. The nuclides vary in their physical characteristics [13]. For effective diagnosis and therapy, it is preferable to employ the nuclides most suited to the particular disease in question. This is why it is important to think about aspects such as the linker, dosage, medicinal drug to be conjugated with, etc., as well as the nuclides themselves. The same mechanism of action underlies all forms of nuclear medicine therapy in which radiopharmaceutical substances are employed to selectively target cancerous tissue, and radiation is conveyed to individual cells via chemical and/or biological adherence.

There are two main types of particle radiation that are used for medical purposes, namely, radiation with α and β particles, and both of them have a similar feature: destruction of malignant cells as a result of severe DNA detrition. Some isotopes (e.g., ^131^I, ^186^Re, ^153^Sm etc.) can emit both therapeutic β^−^ particles as well as γ rays, making simultaneous diagnosis and therapy possible [14,15,16,17,18,19]. Diagnostic and therapeutic radiopharmaceuticals are referred to as theragnostic pairs when they access the same cellular structure and biological process. It is essential to keep in mind that the majority of the theragnostic pairs that are currently being utilised in nuclear medicine are formed from radiopharmaceuticals only. However, there are also “hybrid” theragnostic pairs that are constructed from a radiopharmaceutical intermediary and a diagnostic or therapeutic element derived from other modalities [20]. In particular, from the theragnostic point of view, treatment according to the correct diagnosis has a higher chance of success than random treatment in cancer therapy [21]. Various theragnostic targeting strategies are being studied as potential techniques for cancer treatment, and are expected to enhance therapeutic efficacy and minimise side effects involving systemic toxicity [22].

**Scheme 1:**
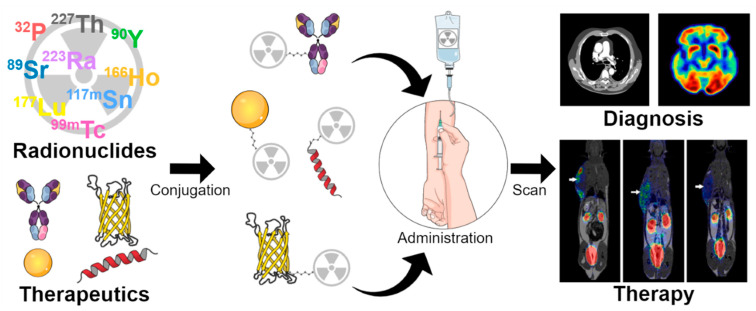
Schematic figure illustrating the use of radionuclides as theragnostics. They have been steadily developed in terms of both diagnosis and therapy.

Radionuclides (radioactive nuclides or radioisotopes) have been studied for the treatment and diagnosis of disease for over 100 years. However, the utilisation of radionuclides in pharmaceutical medicine has faced several problems and limitations, such as the insufficiency of their targeting mechanisms. Iodine-131 is a radionuclide that was used to treat thyroid cancer in 1946, which was a great breakthrough in pharmaceutical medicine [23]. Other attempts have been made to develop radionuclides, such as iodine-131, but they were not very successful. Later, advancements in pharmaceutical medicine improved the use of radionuclides. The newly developed selective targeting delivery of radionuclides can prevent the occurrence of unfavourable delivery, thus reducing their side effects [24]. Moreover, the specific targeting delivery of radionuclides enhances the imaging quality at sites of interest, such as that of the tumour [25,26,27,28,29,30,31]. Targeting moieties such as small molecules, proteins, peptides, and antibodies are commonly utilised for their pharmaceutical applications. Along with them, the use of artificial single-strand oligonucleotide sequences, also known as aptamers (e.g., DNA, RNA, etc.), recently gained significant attention due to their high affinity and specificity when binding with biological molecules. Recently, they have also been employed in radiolabelled imaging techniques such as single photon emission computed tomography (SPECT) and positron emission tomography (PET) [32,33]. From the pool of several techniques for radiolabelled drug delivery, we will be concentrating on peptide-based delivery, which offers several advantages such as an enhanced targeting effect, low systemic toxicity, and easy mass production [34] (Scheme 1). Therefore, peptide–drug conjugates (PDCs) based on radionuclides could represent a promising strategy for clinical application.

As a diagnosis tool, iodine and lutetium radioisotopes co-emit a γ photon, which can be detected by single-photon emission computed tomography (SPECT) [35]. In addition, positron emission tomography (PET) is capable of detecting β^+^ decay with the emission of a positron. Further, radioisotopes can be explored as potential therapeutic agents because they have properties that enable them to emit α^−^ particles, β^−^ particles, and Auger electrons, which can damage deoxyribonucleic acid (DNA) and lead to cell apoptosis [36,37]. Among commonly used radioisotopes in biomedical applications are the short-lived β+ emitters ^15^O, ^13^N, ^11^C, and ^18^F, and the long-lived β^+^ emitters ^89^Zr, ^64^Cu, and ^52^Mn [38]. In the case of α-emitters, ^213^Bi, ^223^Ra, ^211^At, and ^225^Ac have been investigated for the treatment of cancer metastases due to their apoptotic effects [39]. Various theragnostic strategies involving them have been studied, and some radioisotopes, such as ^225^Ac, have been utilised for cancer therapy in preclinical and clinical trials [40,41,42,43].

While discussing the signal detection, one of the well-established methods for maximising the sensitivity is pre-targeting that enables a clear distinction between targets and non-targets [44]. In a nutshell, the first step is to provide drugs that have a very high level of target specificity (different disorders such as tumours, malignancies, etc.). After sufficient binding has occurred between the target substance and the target, nuclides, which have very strong specificity for the target substance, are administered. This technique not only causes more nuclide accumulation in the target, but free nuclides that remain in the blood and tissue are removed for a sufficient amount of time, reducing background and noise significantly. Furthermore, the unlabelled antibody can be eliminated with a clearing agent, and then the nuclide-labelled antibody can be administered sequentially [45]. Finally, pre-targeting can be used for the development of more sensitive and accurate theragnosis. A recent study developed trans-cyclooctene-functionalized amyloid-beta-specific antibodies for the diagnosis of Alzheimer’s disease [46]. ^64^Cu was used as a nuclide; it was rapidly and accurately coupled with an amyloid-beta-specific antibody via click chemistry, and amyloid-beta was well detected using a targeting technique.

In this review, we aim to highlight the recent advancements in the use of radiolabelled peptides for cancer diagnosis and treatment, as well as to provide a brief account of the chemical and biological pathways of complex systems and their smooth transitions into clinical application. From a theragnostic point of view, the utilisation of peptides with radionuclides has various potential functions for clinical applications. Functional peptides also provide interesting features to radionuclides, allowing them a remarkable tumour-targeting capacity, low toxicity, and a strong anticancer effect. Additionally, peptide-based radionuclides commonly used for theragnostic and their mechanisms of action are described through interesting examples of the fabrication of radiolabelled peptides. Finally, we summarised the use of various radiolabelled peptides for cancer theragnosis and its association with clinical aspects.

## 2. Various Radionuclides for Theragnosis

**Figure 1:**
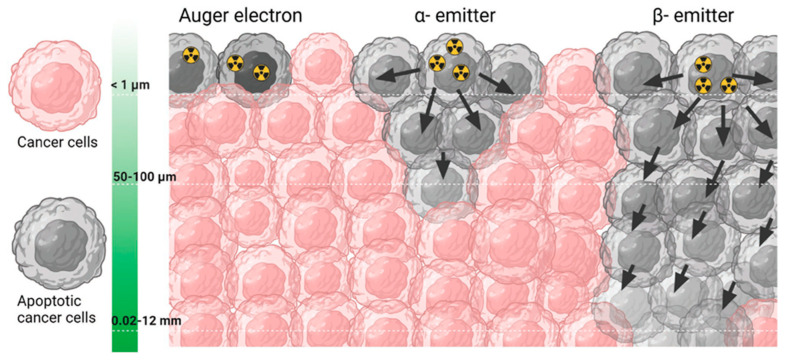
Damage caused by radioactive emissions from radionuclides, Auger electrons, α-emitters, and β-emitters. Although Auger electrons and α-emitters can cause DNA damage, the latter exhibit high-density ionization effects. β-emitters cause repairable damage to single-strand DNA. Reproduced with permission, copyright JOHN WILLEY AND SONS [48].

Diagnostic biomarkers are often coupled with therapeutic medicines because they share common targets in cancer cells or tissues. Radioactive tracers or radionuclides are the pillars of this theragnostic concept, which is also employed in precision oncology. Nuclear theragnostic agents can deliver ionization and radiation to affect tumour cells or tissue, allowing for the imaging of the targets with the use of radioactive substances. In general, radiation with α and β particles is used in nuclear medicine, as both can cause severe damage to the cells due to their ability to destroy DNA (Figure 1). Another class of radiotherapy uses Auger electrons, which are electrons with very low energy emitted by radionuclides. The energy that is placed in nanometre–micrometre reserves induces high linear energy transfer (LET). For this reason, when it is discharged in a close propinquity to cancer cells, it can cause immense impairment by attacking DNA, both unswervingly and meanderingly through water radiolysis [47]. These radioisotopes, which are used in medicines, are studied thoroughly before being incorporated into clinical trials.

Holmium-166 (^166^Ho)-labelled microspheres with high activity are utilised in interventional radioembolization, along with SPECT (single-photon emission computed tomography) imaging, for dosimetry purposes. The ensuing high count rate may have an impact on dead time, lowering the dosimetric precision and image quality [49]. High purity ^166^Ho radioisotopes can be synthesised by one neutron activation of the ^165^Ho isotope. The ^166^Ho isotope emits two very high-energy β particles (1774.32 keV, yield 48.8%; 1854.9 keV, yield 49.9%) and γ rays (80.57 keV, yield 6.7%; 1379.40 keV, yield 0.9%). Due to its high energy, it can be visualised using a gamma camera when injected into the body [50]. ^166^Ho is one of the most promising radionuclides for theragnostic use due to its relatively high specific activity and short physical half-life. For example, [1,4,7,10-tetraazacyclododecane-1,4,7,10-tetrayltetrakis(methylene)]tetrakis(phosphonic acid) (DOTMP) labelled with ^166^Ho strictly localises in the skeleton and delivers a therapeutic dosage to the adjacent bone marrow. As multiple myeloma is primarily contained in the marrow and has a steep dosage response to radiation, it can be used as a therapeutic for bone marrow treatment in patients with multiple myelomas [51,52]. Bahrami-Samani et al. studied ^166^Ho as a therapeutic, using ^166^Ho-ethylene diamine tetra(methylene phosphonate) (EDTMP) for metastatic bone pain palliation [53]. Because ^166^Ho is highly paramagnetic, it can be used for both scintigraphy and magnetic resonance imaging (MRI), making it possible not only to determine the extent of a surgical disease before the procedure, but also to evaluate the biodistribution through quantitative analysis [54,55].

Furthermore, ^166^Ho can be applied in theragnosis by combining it with various polymers. A relatively well-established study by Ha et al. focused on the ^166^Ho-chitosan complex and evaluated its theragnostic effect in 22 cystic brain tumour patients [56]. In this pilot study, 70% of the patients had a radiological response. In addition, the therapeutic effect of the ^166^Ho-chitosan complex was observed in both low-grade astrocytoma and benign tumours. These results suggest an improvement in the clinical evaluation of the group that was administrated ^166^Ho-chitosan. Therefore, the ^166^Ho-chitosan complex could be used as a promising theragnosis for hepatocellular carcinoma. Sohn et al. performed a phase 2 clinical study on 54 hepatocellular carcinoma patients. In this study, ^166^Ho-chitosan showed remarkable results concerning non-fatal toxicities (tolerable toxicities), with a radiological response in 78% of the patients [57].

Lutetium-177 (^177^Lu) is a special nuclide that has gamma and beta co-emitting properties [58]. This particular property is what makes ^177^Lu a promising cancer theragnostic. The emission of gamma rays by ^177^Lu allows for a visualization of the drug’s distribution in the body due to its low energy [59]; this is called scintillation. As a moderate energy beta emitter, ^177^Lu has been reported to be more effective in treating small tumours than other nuclides (especially Yttrium-90, which is a stronger energy beta emitter) [60]. One of the important factors in applying nuclides to patients is the half-life. When the half-life is too long, the nuclide stays in the patient’s body for a long time, increasing the time required for hospitalization and treatment [61]. A long half-life can also cause other side effects, including long-term toxicity, which has not yet been well-studied. In contrast, when the half-life is too short, the distance between the reactor that produced the nuclide and the medical professional assigned to administer it to the patient must be close. This is also rather negative in terms of treatment cost and effectiveness. The physical half-life of ^177^Lu is 6.73 days, which makes it suitable in terms of treatment efficiency, cost, and convenience for patients and medical professionals [59].

Samarium-153 (^153^Sm) is one of the nuclides that produces high radio-nuclidic purity through the neutron bombardment of isotopically enriched ^152^Sm_2_O_3_ [62]. ^153^Sm decays into a stable daughter nuclide, which is ^153^Eu, which has a half-life of 1.9 days [62]. ^153^Sm also has co-emitting properties with beta particles (E_max_ = 705 keV, 635 keV) and gamma photons (103 keV), which are suitable not only for therapy but also for SPECT imaging [63]. ^153^Sm-EDTMP (Quadramet^®^, Lantheus Medical Imaging, Inc., Billerica, MA, USA) has been approved by the US FDA as an excellent radionuclide for bone metastasis. It is a chelating complex of ^153^Sm with EDTMP and is used to reduce pain when cancer metastasises to the bones. It is injected into a vein and distributed throughout the body, and it is preferentially absorbed in the area where cancer has invaded the bone. ^153^Sm-EDTMP’s recommended dose of 37 MBq kg^−1^ has been shown to reduce pain in 55–70% of evaluated patients [64]. The results of one study demonstrated some side effects whereby ^153^Sm-EDTMP led to myelotoxicity and a decrease of 10–40% in platelets and leukocytes. However, all of the patients fully recovered from the side effects after 6–8 weeks.

Yttrium-90 (^90^Y) is one of the most clinically used nuclides due to its unique properties. ^90^Y emits beta particles with 0.937 MeV in energy, and the emitted beta particles can penetrate approximately 2.5 mm of tissue [65]. It is a nuclide suitable for use in transarterial radioembolization (TARE) because it emits relatively high energy and does not penetrate deeply into tissues, resulting in a low risk of side effects. In addition, ^90^Y has a short half-life (2.675 days) [66]. Due to these properties, it has been widely studied and used as a cancer theragnostic, especially for liver cancer [67]. To date, two products have been commercialised in the medical field: glass TheraSphere and resin SIR-Spheres [68]. Selective internal radiation therapy (SIRT) using ^90^Y has been studied for the treatment of advanced hepatocellular carcinoma that cannot be surgically resected [69]. A total of 97 patients (90 males, mean age 60.4 ± 12.3 years) diagnosed with hepatocellular carcinoma that could not be surgically resected underwent selective internal radiation therapy with ^90^Y microspheres. This treatment led to the improvement of all prognostic factors upon administration to the patients in the study. Therefore, selective internal radiation therapy using ^90^Y can be considered a promising hepatocellular carcinoma treatment, especially in patients with combined portal vein tumour thrombus [70].

Of the aforementioned radionuclides, several are involved in the radio-medicine market, as shown in Table 1.

## 3. Radiopharmaceuticals with Radiolabelled Peptides towards Cancer Therapy: Mechanistic Pathway and Biological Paraphernalia

**Figure 2:**
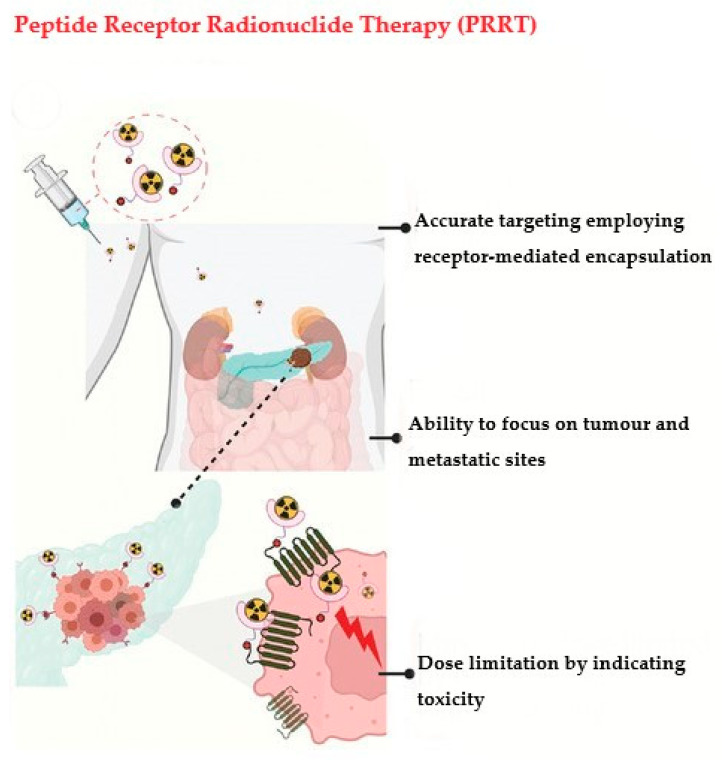
Pictorial representation of peptide receptor radionuclide therapy (PRRT) for pancreatic cancer. PRRT is employed and designed by targeting specific moieties so that selectivity is much higher. Adapted from [48], copyright JOHN WILLEY AND SONS.

The neutralization of cancer cells by radiolabelled peptides follows the radiation-induced killing pathway in therapy. Radiopharmaceuticals are one of the most important tools for fighting cancer, and the development of peptide receptor radionuclide therapy (PRRT) depends on the knowledge gathered from radiotherapy [90]. The mechanistic pathways of radiopharmaceuticals show that some important parameters need to be addressed, such as the complete measurement of physiological and biological functions at the targeted sites (Figure 2) [48]. PRRT solely depends on the process of amassing radiopharmaceuticals at the definite site, which is regulated by several biological processes such as the transference of biochemical species and enzymatic exchanges.

### 3.1. Radiation Dosimetry

In radiotherapy, dosimetry plays a crucial role as it reports the biological data regarding the dose absorbed by both the tumour and the normal tissue. Patient-specific dosimetry depends on the time-ordered distribution of radiopharmaceuticals, and imaging techniques are used for internal dosimetry evaluation. When the radionuclide sits on the surface of cancer cells, a small amount of released energy is deposited into the target cells [91]. This is somewhat countered by the higher concentration that may be achieved in tiny clusters of cells as compared to large, quantifiable tumours. Different techniques can be used to obtain data on the distribution of radiopharmaceuticals in a patient, which include single-photon emission computed tomography (SPECT) and positron emission tomography (PET) [92], as well as whole-body emission counting [93] and planar γ-imaging [94]. Several dosimetry methods have been methodically and rigorously researched and developed. Among them, we will discuss Monte Carlo simulation, S values, local energy deposition, and dose kernels.

Monte Carlo (MC) simulation is a statistical method that determines the three-dimensional interactions of radioactive particles that involve a random pathway [95]. Considering tissue penetration depth, energy loss, bremsstrahlung photons, and cross-fire dosage, the MC model is typically detailed. The key benefits of MC simulations include their capacity to take into consideration an inhomogeneous radioactivity distribution, patient-specific organ geometries, induction of secondary particles (typically γ-radiation), and transitions between tissue types [96]. From measurement-based calculations to pencil beam algorithms to superposition/convolution algorithms, the dose calculation engines used for treatment planning in radiotherapy have continuously improved in terms of accuracy. Monte Carlo treatment planning (MCTP) was first developed in the 1990s, after the successful implementation of MC codes to derive patient-specific dose distribution data, but the main obstacle to efficacious clinical enactment has always been computer power [97]. Owing to its computing approach, MC simulations are very time-consuming; hence, commercial MC software includes several approximations in order to reduce the simulation time, which affects the accuracy. This is why MC simulations are not prioritised over most analysis-based algorithms for treatment planning [98].

The Society for Nuclear Medicine’s Medical Internal Radiation Dose (MIRD) committee devised a way to calculate the average radiation doses received by patients through radiopharmaceuticals. When utilising S values, as specified in MIRD brochures no. 5 and no. 11, it is presumed that radioactivity is distributed uniformly within organs, and that organ mass is standardised [99]. In the past, dosimetry analysis has relied on straightforward mathematical humanoid models that assume the presence of infinite homogeneous fluids with soft tissue density and include spheres of various volumes. The most recent voxel-based anthropomorphic phantoms from the MIRD/ICRP (International Commission on Radiological Protection) are designed for men, women, and children of various ages. Although diagnostic imaging may be used to create patient-specific organ masses, it is currently not possible to modify the location, tissue inhomogeneity, or form of the organs [100,101]. Relatively quick and easy techniques that only require consecutive 2D imaging to estimate activity distributions and the use of average organ characteristics have made S value dosimetry suitable for clinical usage. For this reason, this method is now accepted as the norm for dosimetry in pharmaceutical research.

In molecular radionuclide therapy, dose kernel convolution (DK) algorithms have been proposed in order to expedite estimations of the absorbed dosage. Voxel-based dosimetry using voxel S values (VSVs) and MIRD formalism is provided in MIRD brochure no. 17 (1999) [102]. VSVs are determined using MC simulations and given for certain isotopes and voxel dimensions. The capacity to handle irregular radioactivity dispersion at the organ or tumour level is a virtue of dose kernel dosimetry. Furthermore, in the assessment of radiobiological repercussions, 3D dose distributions facilitate the depiction of dosage lines and dose–volume histograms (DVHs). Dose kernel research focuses on density adjustments and techniques for calculating and comparing several kernels more quickly [103,104,105].

The aforementioned techniques are recognised as the pillars of radiation dosimetry, and along with them is the local energy deposition method. In this method, it is assumed that all energy is absorbed in the voxel of origin [106]. Due to the longer penetration depth, this theory does not apply to γ-emissions or secondary photons, but it does hold for some α and β particles and Auger electrons. Nevertheless, this approach is relatively accurate for a rapid study, such as in genotoxicity, if one is only seeking to investigate specified sections of the radionuclide emission spectrum [107,108,109].

### 3.2. Localization Pathways

Molecular imaging and treatment with radioisotope-labelled molecules, also called radiopharmaceuticals, depend on the molecules’ ability to target diseased cells. In this section, we will briefly explore the different approaches to radiopharmaceutical localization that are used in diagnostic imaging and medical treatment.

The term “passive diffusion” is used to describe the random movement of molecules from areas of greater to lower concentration in order to achieve homogeneity. However, in a living system, this kind of motion often involves molecular transport across a membrane. Molecule mobility across membranes is affected by factors such as pH, ionization, molecule size, and lipid solubility [110]. Since phospholipids, glycolipids, sphingolipids, and sterols are the prevalent types of lipids that make up membranes—of which phospholipids are the main component—lipid solubility is the main decisive factor. As a result, only molecules that are soluble in lipids (known as lipophilic molecules) can pierce through membranes; polar hydrophilic ones cannot. The membranes prevent the diffusion of lipophobic molecules while permitting the passage of lipophilic ones. This barrier can be broken as a result of some physiological disorders, which then allow hydrophilic molecules to diffuse into the brain tissues. ^99m^Tc-DTPA usually follows this kind of localization mechanism when used for brain imaging. Due to its hydrophilic nature, it cannot usually cross the barrier, but when abnormalities arise which create a perturbation across the blood–brain barrier (BBB), using this passive diffusion process ^99m^Tc-DTPA crosses the barrier [111,112,113,114].

A capillary blockade relies on microembolization (the trapping of radiolabelled particles in the capillary bed), which is key to the accuracy of this method; it is used to calculate the perfusion of organs such as the brain, heart, and lungs. Somatostatin analogues and annexin V tagged with ^64^Cu, ^68^Ga [115], or ^99m^Tc follow this kind of mechanism.

Active transport is the metabolic, energy-requiring route by which a carrier mediates the transfer of a radiopharmaceutical across cellular membranes. ATP provides the necessary energy for this process, allowing molecules to be transported across a gradient of concentrations. Saturation, the maximal response offered when all the carriers are engaged, is achieved due to the carrier-selective nature of the system, which explains the fitting of a small number of molecules into a specific carrier [116].

Compartmentalised localisation is the process by which one or more preferred species are disseminated inside a limited area. In a radiopharmaceutical context, “compartment-localisation” refers to the process of confining a radiotracer within a defined volume and maintaining it long enough to allow for a thorough examination of the volume. Under normal conditions, the fluids in the body’s compartments flow in a systematic pattern, but pathologic changes can disrupt this flow, producing abnormalities. In human anatomy, the vascular system, cerebrospinal fluid space, peritoneal cavity, etc., are designated as biological compartments. ^111^In-DTPA imaging is used to monitor the leakage of CSF [117], which is attributed to the mechanistic pathway of unexpected leaking from its designated repository caused by pathologic alterations.

The specificity and efficacy of the tracer agent can be quantified by the intensification of radiopharmaceuticals in afflicted cells. Along with the aforementioned pathways, some others are also in operation, including phagocytosis [118], ion exchange [119], filtration [120], cellular migration [121], and facilitated diffusion [122]. The success of radiopharmaceuticals depends on their accumulation in specific target cells, expressed as a percentage of the amount that was injected into the organ or tissue.

## 4. Fabrication of Amino Chains for Radiopharmaceutical Applications

**Figure 3:**
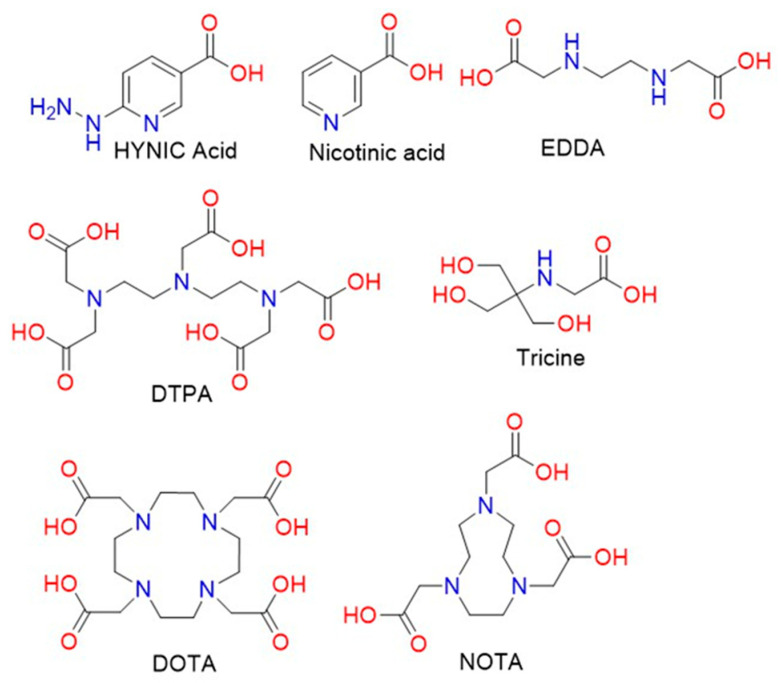
Structural formulae of different chelators and co-ligands for the radiolabelling of peptides and proteins.

There is an increasing need to develop advanced and sophisticated procedures for successful delivery to target sites. With advances in chemistry, chelation has been chosen as a potential method for the insertion of metallic cations (e.g., ^177^Lu ^+3^, ^90^Y ^+3^, etc.), considering the rate of metal complex formation and dissociation. The size of the cavity of bifunctional chelating agents (BFCAs) and the ionic radius of the cationic metal should be compatible in terms of metal binding with high stability and limiting dissociation [123]. Additionally, to circumvent the possible interference between the active site of the chelator and the receptor-binding site, a linker may be required [124]. Commonly used linkers such as PEG and amino chains have been utilised as pharmacokinetic modifiers. Many well-designed BFCAs have been explored for the fabrication of radiolabelled peptides. The common structures of acyclic and cyclic BFCAs for the development of radiolabelled amino chains are shown in Figure 3 [125].

**Figure 4:**
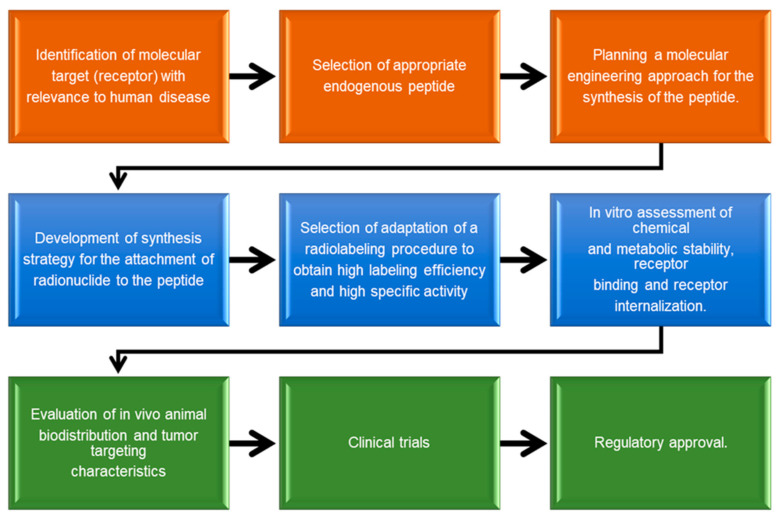
Illustration of major strategies involved in the preparation of peptide-based radiopharmaceuticals.

Chelators such DTPA, DOTA, and NOTA or derivatives are often used for radiolabelled peptides. An unstable chelation process involving radionuclides and chelators leads to possible interactions such as trans-chelation to blood proteins and enzymes. Therefore, the selection of chelating agents and their optimal interaction with metallic ions are crucial factors for the fabrication of peptide-based radiopharmaceutical applications [126]. BFCAs can be introduced to peptides via some bioconjugation methods such as amide bonding, thiol coupling, and oxime bond formation, as well as click reactions. More interestingly, some important regulatory peptides are currently of interest for the development of radiolabelled peptides, which target receptors that are overexpressed in tumour cells. Peptide analogues may act as receptor agonists or antagonists while delivering radionuclides to targeting sites [127]. Peptides and their radiolabelled analogues function as agonists when they are internalised by receptor-mediated endocytosis, leading to the accumulation of radioisotopes in tumour cells. We have illustrated the strategies for developing peptide-based radiopharmaceuticals below [128] (Figure 4).

## 5. Radiolabelled Peptides Used in Cancer Theragnosis

A variety of receptors involved in hormone regulation are overexpressed in a wide assortment of human malignancies; thus, an effective alternative to hormone replacement therapy is a peptide-based strategy for recognizing theragnostics that target tumours. The use of peptides to administer cytotoxic radiation to tumours through binding with overexpressed receptors is known as radionuclide therapy (RNT) or peptide receptor radionuclide treatment (PRRT). Interest in the targeting of tumours with radioactive peptides has been emerging, chiefly prompted by OctreoScan’s breakthrough in the early 1990s, wherein somatostatin receptor subtype 2 or SST-2-positive tumours were identified. Since then, peptides have been utilised to strategically target tumours by binding them with an eclectic assortment of overexpressed receptors. In this section, we will discuss recent advancements in radionuclide–peptide-based theragnostic targeting.

### 5.1. Somatostatin Receptor-Targeted Anticancer Therapy

**Figure 5:**
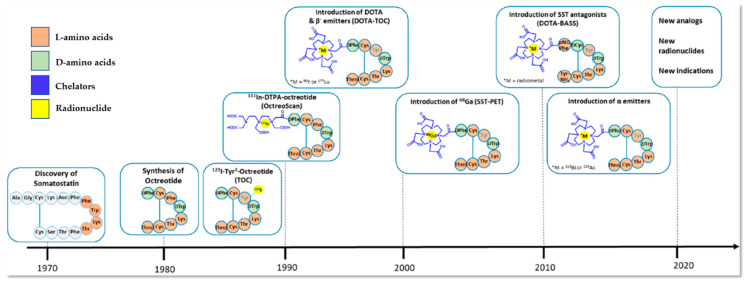
Brief timeline of somatostatin receptor-targeted peptide evolution. Adapted from [137]. Discovery of the somatostatin analogue and its evolution and transition into treatment strategy.

Somatostatin, a cyclic peptide, is a significant physiological modulator of neuroendocrine activity in various organ systems across the human anatomy. Its activity is mediated by five distinct SST receptor subtypes (SSTR1–5), and it inhibits the release of hormones as well as the growth of tumours. SSTR subtype expression fluctuates amongst various pituitary adenomas and tumours that secrete the same hormone [129]. Neuroendocrine tumours (NETs) are a category of malignancies that develop from neuroendocrine cells that are widely spread throughout the body and share similar characteristics with both endocrine (hormone-producing) and nerve cells (neurons) [130]. Numerous neuroendocrine cells, such as anterior pituitary somatotroph, thyroid C, and pancreatic islet cells, have been shown to express somatostatin receptors [131,132]. Among them, SST-2R is typically overexpressed in NETs, thus allowing for the development of theragnostics that selectively target SST-2R-positive NETs. This was the crucial piece of information that facilitated the first successful imaging of NETs, which was carried out by E.P. Krenning and colleagues [133]. They developed the first radiolabelled somatostatin analogue, namely, ^123^I-labelled Tyr^3^-octreotide. By 1990, it had been used on hundreds of patients in clinical trials, producing fine single-photon emission computed tomography (SPECT) images of localised carcinoid tumours, paragangliomas, and pancreatic endocrine tumours. However, the high biliary excretion of ^123^I resulted in a concentrated colonic accrual, which disrupted the construal of planar and SPECT images of tumours in the abdomen. This paved the way for the discovery of diethylenetriaminepentaacetic acid-d-phenylalanine (DTPA)-octreotide radiolabelled with indium-111 (^111^In-DTPA-D-Phe^1^-octreotide) [134], which showed high sensitivity towards the localisation of NETs. This work was published by the same group and involved more than 1000 patients, resulting in a landmark for nuclear medicine known as “The Rotterdam Experience” [135]. In 1994, the FDA approved this based on 350 European patients’ data, which showed higher sensitivity and specificity towards gastroenteropancreatic-neuroendocrine tumours (GEP-NETs) as compared to CT and MRI, making it the first FDA-approved radiopharmaceutical imaging agent (Figure 5). This is commercially available as OctreoScan™. It is successfully used in many common types of tumours, including carcinoid tumours, islet cell tumours (particularly gastrinomas, glucagonomas, and VIPomas), small cell lung cancer, pheochromocytoma, paraganglioma, and pituitary adenoma [136].

Another commercially available SSTR analogue named ^99m^Tc-depreotide (commercial name: NeoTect), which binds with SSTR subtypes 2, 3, and 5, is used to treat patients with non-Hodgkin’s lymphoma. Following the path of Octeroscan ™, this functions in the same way by identifying the lymphoma sites. Due to its in vivo half-life, optimal biodistribution, and high binding affinity, Depreotide (cyclo-[(N-Me)Phe-Tyr-D-Trp-Lys-Val-Hcy]CH_2_-CO.β-Dap-Lys-Cys-Lys.amide, P829) labelled with a beta emitter was developed as a tumour-imaging radiopharmaceutical by D.L. Bushnell and colleagues [138]. In addition, Hossein Behnammanesh and colleagues reported a ^177^Lu-labelled somatostatin receptor for the targeted therapy of NETs. After effective computational evaluation of the ligand, they successfully synthesised ^177^Lu-DOTA-p-Cl-Phe-Cyclo(D-Cys-L-BzThi-D-Aph-Lys-Thr-Cys)-D-Tyr-NH_2_ (^177^Lu-DOTA-Peptide 2). They examined its stability, receptor binding, biodistribution, and SPECT imagery using C6-tumour-bearing rats. In both tumour-bearing and normal rats, the radiopeptide’s pharmacokinetics demonstrated rapid blood clearance, high pancreatic absorption, and no discernible retention in the liver. For the in vitro and in vivo tests, glioma C6 cells naturally expressing the SSTRs 1, 2, 3, and 5 were used. From the SPECT/CT imaging of C6 tumour-bearing rats, the in vivo interaction capability of ^177^Lu-DOTA-Peptide 2 was confirmed and compared with tumours blocked with octreotide [139].

Among other somatostatin analogues, ^177^Lu-DOTATATE is one of the most widely used PRRTs globally. Surprisingly, when a dosimetric study was performed by introducing an albumin-binding moiety, Evans’ Blue, ^177^Lu-DOTATATE showed remarkably higher uptake and retention in NETs [140]. Based on the pharmacokinetic profile, it was evident that 2 h after the injection of ^177^Lu-DOTA-EB-TATE (^177^Lu-1, 4, 7, 10-tetra-azacyclododecane-1, 4, 7, 10-tetraacetic acid-Evans blue-octreotate), there was high accumulation in the blood as well as a moderate uptake in the liver, spleen, and kidneys; however, in the case of ^177^Lu-DOTATATE, there was surprisingly no blood accumulation detected [141]. Using the same radiolabelled ligand, ^177^Lu-DOTA-EB-TATE, another study reported its therapeutic efficacy and safety. The research concluded that ^177^Lu-DOTATATE is effective towards NET treatment, but fast blood clearance creates an obstacle as the delivered radiation to the NETs was rather low, whilst ^177^Lu-DOTA-EB-TATE showed significantly higher tumour uptake [140].

Although ^177^Lu-DOTATATE PRRT is a well-established therapeutic for cancer patients, in some cases, patients who are refractory or stable in response to that treatment regimen can experience complications. A new study has reported the use of ^225^Ac-DOTATATE-targeted alpha therapy on GEP-NET patients who are ^177^Lu-DOTATATE PRRT-resistant. The study showed that following two cycles of ^225^Ac-DOTATATE therapy, 62.5% of patients showed partial phenotypic remission which lasted long enough to have an anti-tumour effect during the study assessment, whereas the rest showed disease stability. The toxicity was evaluated, and no further haematological toxicity was found; likewise, no significant variance in haemoglobin, blood urea, serum and creatinine levels, thrombocytopenia, neutropenia, or lymphopenia was observed. Only a decrease in the platelet count was documented, along with nausea, loss of appetite, and vomiting. The researchers concluded that the short duration of follow-ups can falsely predict long-term outcomes such as overall survival, enduring anti-tumour response length, etc., indicating that multicentre randomised controlled trials are needed [142].

**Figure 6:**
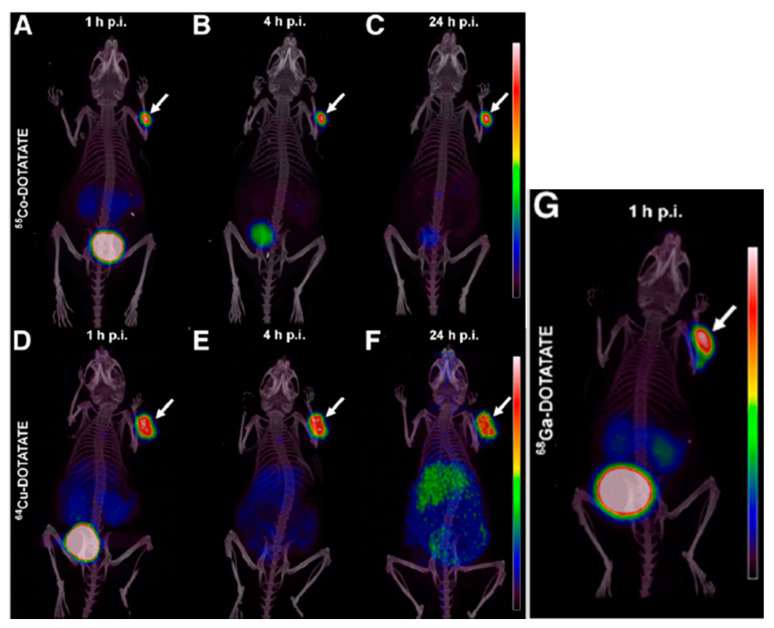
(**A**–**C**) [^55^Co]Co-DOTATATE, (**D**–**F**) [^64^Cu]Cu-DOTATATE, and (**G**) [^68^Ga]Ga-DOTATATE scans. Adapted from [143].

In addition, an important study published by Thomas L. Andersen and colleagues compared [^55^Co]Co-DOTATATE, [^64^Cu]Cu-DOTATATE and [^68^Ga]Ga-DOTATATE to in vivo imaging characteristics in an SSTR-positive xenograft mouse model. The capacity of PET imaging to improve picture contrast and, hence, the detectability of cancers is dependent not only on the discovery of new targeting mechanisms with new tracers, but also on the imaging properties of the radionuclide. Figure 6 depicts the maximum intensity projection from PET/CT scans with different radio tracers. It clearly shows the higher relative liver uptake observed for the ^64^Cu tracer and the higher bladder uptake for ^68^Ga [143].

While investigating the therapeutic potential of somatostatin analogues labelled with β-emitters, along with ^177^Lu, ^90^Y was also taken into consideration. Being a high energy β-emitter, its minimal deep tissue penetration abilities remove critical side effects. Back in 2001, Marion de Jong and colleagues reported a tumour response involving ^90^Y-labelled octreotide radionuclide therapy. They concluded that their experiment and the efficacy of their therapy was totally dependent on the tumour size [144]. From their experiments, it was evident that ^90^Y showed greater efficacy on large tumours, while ^177^Lu was better for small ones. This was one of the key moments in ^90^Y-labeled radiotherapy which paved the way for modern clinical protocols of PRRT using yttrium. The same group of researchers published another report of in vivo PRRT results involving Tyr^3^ -Octreotide and Tyr^3^ -Octreotate radiolabelled with both ^177^Lu and ^90^Y, using tetra-azacyclododecatatro-acetic acid (DOTA) as a chelator. In both of the cases, they successfully controlled the tumour size in a dose-dependent manner. Although ^90^Y has higher energy compared to ^177^Lu, this study found that absorbed energy per cell was lower for yttrium compared to lutetium [145].

Back in 2020, Anna Zemczak and colleagues reported a safety and efficacy of repeated PRRT with [^90^Y]Y/[^177^Lu] Lu-DOTATATE in patients with NETs. They achieved a high disease control rate of 92.3% after repeated PRRT and they concluded from their research that their protocol and treatment regime was effective for patients with inoperable G1 and G2 neuroendocrine tumours [146]. Recently, in 2022, an international multicentre study was published regarding ^90^Y radioembolization (RE) treatment for neuroendocrine neoplasms (NENs) by Benedikt M. Schaarschmidt and colleagues. From their findings, it was evident that patients who had undergone ^90^Y RE as a second line of treatment did show a significant increase in overall survival, as well as hepatic and global progression-free survival. The requirement that PRRT only be used on patients with elevated somatostatin receptor expression relative to baseline hepatic uptake does not hamper ^90^Y RE [147].

Due to the critical-organ dose barrier, delivering a sufficient tumour radiation dose is still a challenging factor. Keeping this in mind, a new combination method for delivering PRRT was introduced by David L. Bushnell and colleagues in which they used ^131^I-metaiodobenzylguanidine (^131^I- MIBG) to PRRT involving ^90^Y-DOTATOC. While comparing the results between combination and single ^90^Y-PRRT, they did not find any dose-limiting toxicities in either case, while tumour dosimetry evaluated a dose increase of 34–83% using combination therapy [148].

Radiolabelled somatostatin analogues are currently being used for therapy, with great affinity to the most widely expressed SST-2. Five somatostatin receptor subtypes (SST1-5) have been documented to express and co-express in a wide variety of patterns according to the tumour type [149]. A different subtype may occupy tumour regions devoid of the expression of a given subtype. Furthermore, in advanced illness stages, the deletion or downregulation of SST2 is linked to a poorer prognosis, less sensitive imaging, and unsuccessful therapy with SST2-specific analogues due to poor tumour targeting [150]. For this reason, somatostatin analogues with an affinity for several receptor subtypes are of considerable interest since they can tackle the problems of receptor subtype co-expression and heterogeneous expression patterns. Targeting a wider variety of tumours or increasing absorption in a single tumour should be possible with analogues that target more subtypes than SST2, making this an area worth investigating.

### 5.2. CD13-Targeted Anticancer Therapy

Neo-angiogenesis in the tumour stroma recruits new blood vessels from the prior vasculature. This is a multi-step process that involves growth factors, adhesion molecules, and cellular receptors, and it is crucial for tumour cell survival, proliferation, and invasion [151]. CD13, a zinc-dependent membrane-bound ectopeptidase, plays a critical role in angiogenesis [152]; it is usually overexpressed in lung, breast, and prostate cancer. Therefore, a significant amount of research has been dedicated to determining the biomarkers that could interact with CD13 that is overexpressed in angiogenic vessels.

Back in 2000, Renata Pasqualini and colleagues first demonstrated aminopeptidase N (APN, also known as CD13) as a receptor for tumour-targeting peptides. They showed that asparagine–glycine–arginine (NGR)-containing peptides specifically bind to the immunocaptured CD13 [153]. This was one of the first steps towards NGR-based peptide development in anticancer theragnosis. In order to identify responsive patients, it was crucial to develop a method for the non-invasive monitoring of CD13 receptor levels in humans. NGR could be a prospective target in cancer therapy based on its determination of the expression of CD13 receptors in vivo during NGR-based molecular radio imaging.

Another research work was published in 2020 by Adrienn and colleagues in which the ^68^Ga-NODAGA-c(NGR) [^68^Ga-c[Lys(1,4,7-Triazacyclononane,1-glutaric acid-4,7-acetic acid)-Asn-Gly-Arg-Glu]-NH_2_] peptide was used to evaluate the efficacy of in vivo molecular imaging. The aim of that research was to determine the anti-tumour effects of bestatin and actinonin treatment in subcutaneously transplanted HT1080 and B16-F10 with the use of tumour-bearing animal models. Five days after injecting bestatin and actinonin, the PET scans showed that bestatin- and actinonin-treated B16-F10 tumours exhibited significantly low radiopharmaceutical uptake and accumulation. This means that the CD13 inhibitor may be suitable for suppressing the neoangiogenic process [154]. A comparative study of monomeric ^68^Ga-NODAGA-NGR and dimeric ^68^Ga-NODAGA-NH_2_ was published by Ina Israel and colleagues in 2021. They aimed to evaluate the target affinity of both against CD13 [155]. Biodistribution data showed that the monomer was more concentrated in most tissues than the dimer, except for the liver and spleen. This information points to the dimer being slightly more hepatobiliarily excreted. However, both NGR peptides were mostly flushed out of the body via the kidneys. According to Alessandra Graziadio and colleagues [156], the functionalization of the -NH_2_ group leads to a decrease in affinity towards CD13. In their study, they used amine group functionalization and conducted further thorough experimentation with modified NGR peptides in order to determine the binding affinities with CD13. They concluded that both were suitable as radiotracers for non-invasive imaging, although a slightly higher tumour-to-muscle ratio was found in the PET imaging of the dimer.

Barbara Gyuricza and colleagues reported another ^68^Ga-labelled, cNGR-based glycopeptide for in vivo PET imaging. They synthesised two different radiopeptides; one was a lactosamine derivative (3-azido-propyl-β-D-galactopyranosyl-(1→4)-(2-amino-2-deoxy)-β--D-glucopyranoside) for glycosylation, and the other was made using the PEG_4_ moiety for PEGylation. After measuring the ex vivo biodistribution through the PET imaging of CD13 positive receptor expression in B16F10 tumours, it was concluded that both were suitable for PET imaging. PEGylating and glycosylation resulted in enhanced pharmacokinetic properties such as higher tumour uptake as well as a high tumour-to-muscle ratio (T/M) [157].

**Figure 7:**
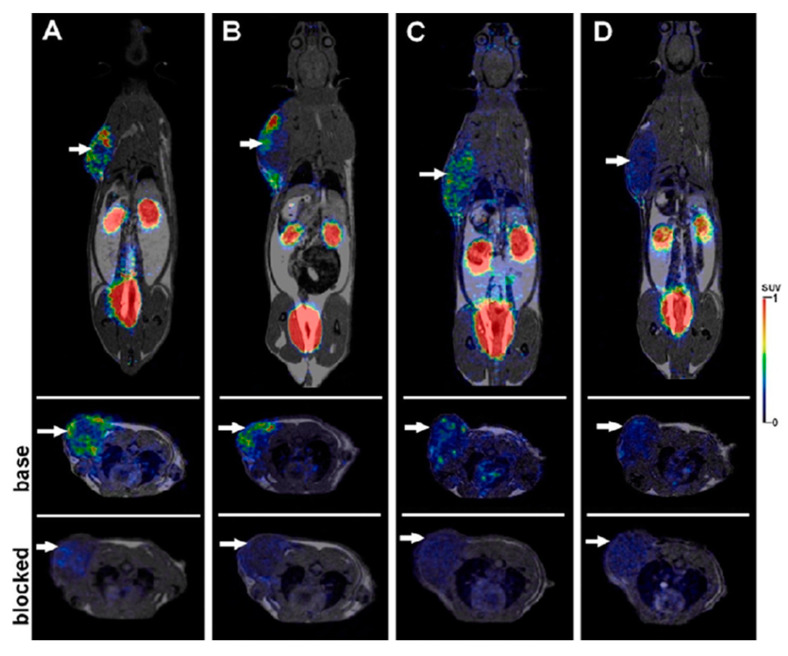
In vivo assessment of ^68^Ga-labelled NGR derivative uptake of He/De tumours via PET/MRI imaging; the upper row represents the coronal region, and the middle and lower rows represent the trans-axial region. The subcutaneously growing tumours were made clearly visible using ^68^Ga-NOTA-c(NGR) (**A**), ^68^Ga-NODAGA-c (NGR) (**B**), ^68^Ga-NODAGA-c(NGR) (MG1) (**C**), and ^68^Ga-NODAGA-c(NGR) (MG2) (**D**). (**A**,**B**) show higher accrual as compared to (**C**,**D**). Adapted from [158], reproduced with permission, copyright Elsevier.

**Figure 8:**
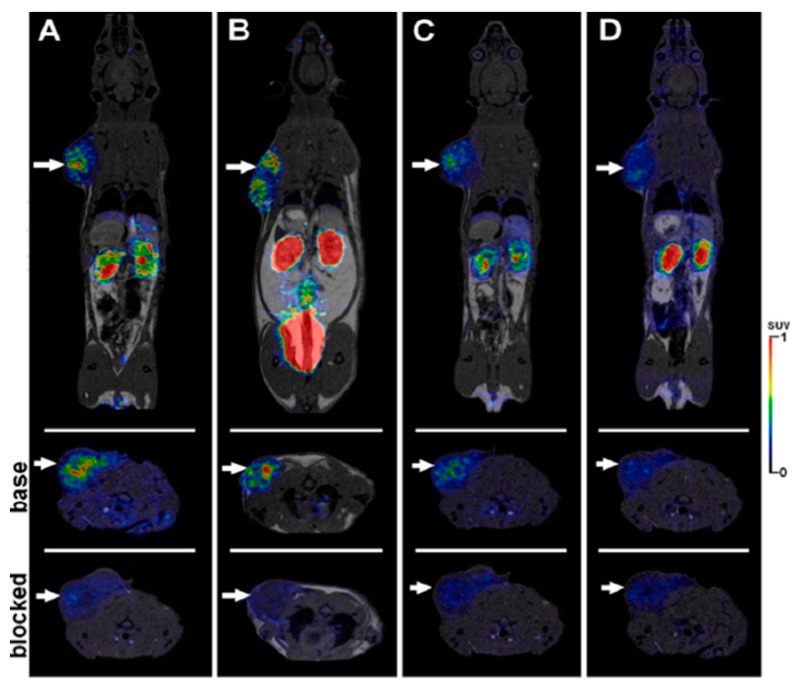
In vivo assessment of ^68^Ga-labelled NGR derivative uptake of Ne/De tumours via PET/MRI imaging; the upper row represents the coronal region, and the middle and lower rows represent the trans-axial region. The subcutaneously growing tumours were made clearly visible using ^68^Ga-NOTA-c(NGR) (**A**), ^68^Ga-NODAGA-c (NGR) (**B**), ^68^Ga-NODAGA-c(NGR) (MG1) (**C**), and ^68^Ga-NODAGA-c(NGR) (MG2) (**D**). (**A**,**B**) show higher accrual as compared to (**C**,**D**). Adapted from [158], reproduced with permission, copyright Elsevier.

Adrienn Kis and colleagues recently published a report containing a detailed in vivo assessment of different ^68^Ga-labelled NGR derivatives in relation to CD13. They synthesised four different cyclic NGR derivatives: ^68^Ga-NOTA-c(NGR) [^68^Ga-c[Lys(1,4,7-triazacyclononane-triacetic acid)- Asn-Gly-Arg-Glu]-NH_2_], ^68^Ga-NODAGA-c(NGR) [^68^Ga-c[Lys(1,4,7-triazacyclononane,1-glutaric acid-4,7-acetic acid;)-Asn-Gly-Arg-Glu]-NH_2_], ^68^Ga-NODAGA-c(NGR) (MG1) [^68^Ga-c[CH_2_-CO-Lys(1,4,7-triazacyclononane,1-glutaric acid-4,7-acetic acid;)-Asn-Gly-Arg-Cys]-NH_2_], and ^68^Ga-NODAGA-c(NGR) (MG2) [^68^Ga-c[CH_2_-CO-Lys(1,4,7-triazacyclononane,1-glutaric acid-4,7-acetic acid)-Asn-N(Me)Gly-Arg-Cys]-NH_2_]. They used radiolabelled cyclic NGR derivatives with the ^68^Ga radiometal in order to assess their APN/CD13 selectivity in vivo through PET/CT imaging in syngeneic hepatocellular carcinoma (He/De) and mesoblastic nephroma (Ne/De) tumour models. They attempted to confirm the presence of neoangiogenic markers in both He/De and Ne/De tumours by PET imaging and with the use of the NGR derivatives. As a result, the He/De and Ne/De tumours were clearly identifiable, but a different accrual was observed. PET imagery and standardised uptake value calculations revealed that NOTA- or NODAGA-conjugated molecules showed higher accumulation as compared to NODAGA conjugated with the use of MG1 and MG2 probes. Figure 7 and Figure 8 below present the imagery and findings [158]. 

Due to its high recurrence and mortality, ovarian cancer is a serious threat to women’s health. It is one of the most common gynaecological malignancies. Most patients with ovarian cancer show advanced intraperitoneal metastasis of the disease at prognosis, owing to its clinically silent nature [159], and the differential overexpression of CD13 has been well-documented [160]. With regard to the expression of APN/CD13, Yi Yang and colleagues reported on the use of the ^68^Ga-labelled dimeric cNGR peptide DOTA-c(NGR)_2_ [DOTA, 1,4,7,10-tetraazacyclododecane-N,N′,N″,N‴-tetraacetic acid] for the micro-PET imaging of ovarian cancer xenografts. A higher uptake of ^68^Ga was found in ES2 cells when compared with SKOV3 cells, which makes their compound a candidate for the assessment of CD13 expression in ovarian cancer [161]. In addition, many therapeutics have been delivered to active tumour neovascular tissue by peptides bearing the NGR motif as a homing agent. However, the affinity, specificity, and pharmacokinetics of radiolabelled NGR peptides from different molecular scaffolds and chelators vary greatly. Peptide dimerization targeting NGRs showed promise for the enhancement of pharmacokinetic properties and the tumour-to-nontarget ratio.

## 6. Radiolabelled Peptides Translated into Clinical Trials

Over the past thirty years, a wide variety of radiolabelled peptides have been smoothly translated into clinical trials. Many of the trials have been completed and are waiting for approval from the US FDA and other medicine approval authorities around the world. In this section, we will be discussing some of the radiolabelled peptides that have been translated into clinical trials.

Metastatic, castration-resistant prostate cancer is one of the most challenging curable diseases. Since castration-resistant prostate cancer does not respond to hormone therapy, there is an urgent need to develop a therapeutic agent that targets this type of cancer [162]. Recently, specific proteins, such as SWI/SNF, which are overexpressed in castration-resistant prostate cancer have been discovered [163]. Prostate-specific membrane antigen (PSMA) is also one of the proteins that is highly expressed in metastatic, castration-resistant prostate cancer [164]. In the clinical trial for the curing of this disease, ^177^Lu-PSMA-617 was investigated as a radioligand therapy that could deliver beta-particle radiation to PSMA-expressing cells and the surrounding microenvironment (NCT03511664) [164]. Oliver et al. evaluated the theragnostic efficacy of ^177^Lu-PSMA-617 through an international, open-label, phase 3 trial involving patients previously treated for metastatic, castration-resistant prostate cancer. The patients had undergone treatment of not only one androgen receptor pathway inhibitor, but also of one or two taxane regimens. Furthermore, PET-CT-scanned patients who used PSMA-positive gallium-68 (^68^Ga)-labelled PSMA-11 also participated. In the clinical trials, one group of patients additionally received ^177^Lu-PSMA-617 treatment (7.4 GBq every six weeks for four to six cycles) as they were provided with protocol-permitted standard care that excluded radium-223 (^223^Ra). The other group of patients only received standard care. The endpoints were determined based on bioimaging of the patients or the objective responses. The side effects that occurred during the clinical trial were limited to those that occurred within 30 days of the last administration of ^177^Lu-PSMA-617 and those that occurred before the subsequent anticancer treatment. According to the imaging-based results for progression-free survival and overall survival, ^177^Lu-PSMA-617 plus standard care led to significant improvement as compared with standard care alone (in the case of imaging-based progression-free survival, median was 8.7 vs. 3.4 months; hazard ratio for progression or death, 0.40; 99.2% confidence interval, 0.29 to 0.57; *p* < 0.001; in the case of survival, median was 15.3 vs. 11.3 months; hazard ratio for death, 0.62; 95% CI, 0.52 to 0.74; *p* < 0.001). However, the incidence of adverse events of grade 3 and above was higher with the administration of ^177^Lu-PSMA-617 (52.7%) than without (38.0%). Fortunately, the adverse events that occurred did not cause negative effects such as a reduction in the quality of life of the patients. Therefore, standard care plus radioligand therapy with ^177^Lu -PSMA-617 as a combination therapy significantly prolonged the imaging-based progression-free survival and overall survival of patients with PSMA-positive, metastatic, castration-resistant prostate cancer.

Other radionuclides, including ^99m^Tc and ^18^F, were studied as theragnostic agents for cancer. Zhenying et al. aimed to investigate the value of ^99m^technetium-three polyethylene glycol spacers-arginine–glycine–aspartic acid ([^99m^Tc]3PRGD_2_) imaging in diagnosis, which can also be used for the staging of breast cancer [165]. [^99m^Tc]3PRGD_2_ was compared with 2-deoxy-2-[^18^F]fluoro-D-glucose ([^18^F]FDG) to evaluate theragnostic properties in integrin α_v_β_3_-expressed tumour vascular endothelial cells. Since α_v_β_3_ is overexpressed in breast cancer, it is a promising target for cancer treatment [166,167,168]. [^99m^Tc]3PRGD_2_ and [^18^F]FDG were investigated in a study involving 42 women who were suspected to have breast cancer [165]. To be able to analyse the breast lesions, [^18^F]FDG imaging was employed, and the tumour–blood (T/B) ratios from [^99m^Tc]3PRGD_2_ imaging and the maximum standardised uptake value (SUV_max_) were measured. These two radiopharmaceuticals showed no significant difference between their area under the curve (AUC) values derived from the imaging for the semi-quantitative analysis (0.880 and 0.955; Z = 0.88, *p* > 0.05). Furthermore, the sensitivity, specificity, and accuracy of [^99m^Tc]3PRGD_2_ and [^18^F]FDG were evaluated for axillary lymph node metastasis. As a result, [^99m^Tc]3PRGD_2_ uptake was correlated with the expression of integrin α_v_β_3_, which was significantly higher in the HER2-positive and stage III-IV patients (*p* < 0.05). With these valuable results, the prospective study demonstrated that [^99m^Tc]3PRGD_2_ imaging could be promising in pharmaceutics, not only for the diagnosis of breast cancer but also for its staging. Because integrin α_v_β_3_ in tumour microvessels is associated with the breast cancer subtype and its staging, [^99m^Tc]3PRGD_2_ could show different sensitivities for the detection of small lymph node metastatic lesions when compared with [^18^F]FDG imaging.

A recent clinical trial suggested the possibility of using ^18^F-labelled radionuclides as an economical theragnostic [169]. The ^68^Ga-labelled somatostatin analogue (SSA) PET is widely known as a promising theragnostic. The current standard for somatostatin receptor (SSTR) imaging is limited by practical and economic challenges. It is worth noting that Elin et al. used fluorine-18-labelled alternatives, such as the recently introduced [^18^F]AlF-NOTA-octreotide ([^18^F]AlF-OC), to overcome the limitations of SSTR. This trial was also performed on neuroendocrine tumour (NET) patients to evaluate the dosimetry, biodistribution, pharmacokinetics, and lesion targeting of [^18^F]AlF-OC. An IV (intravenous) bolus of 4 MBq kg^−1^ [^18^F]AlF-OC was administrated to six healthy volunteers. The healthy volunteers underwent an additional PET/CT scan at 150 and 300 min post-injection, with serial, whole-body PET scans performed from the time of tracer injection up to 90 min post-injection. A high and comparable detection ratio was evaluated for both tracers (86.0% for [^68^Ga]Ga-DOTATATE vs. 90.1% for [^18^F]AlF-OC at 2 h post-injection; *p* = 0.68), with [^18^F]AlF-OC-administrated patients showing favourable kinetic and imaging characteristics. Further validation may be needed for the use of [^18^F]AlF-OC as a fluorine-18-labelled alternative.

## 7. Conclusions and Future Directions

There is an increasing requirement for the development of stable and well-defined radiolabelled peptides or biomolecules for use in targeted cancer therapy. Hence, it is important to develop advanced strategies that use radiolabelled peptides for both clinical evaluation and therapy [170]. The great advantages of peptide-based radionuclide therapy are its ability to accurately target specific sites, low antigenicity, and easy synthesis protocols. However, there is an initial requirement to identify human disease-specific receptors and relevant peptides. The next step can be the conversion of radionuclides into peptides, and it is crucial to design new radionuclides that will retain their receptor binding affinity. Advanced technologies, such as the conjugation of bifunctional chelating ligands to peptides and the incorporation of radionuclides into chelating ligands, have been developed for radiolabelled peptide-based cancer therapy. Some initial assessments, such as the binding of the radiopeptide with tumour cells, stability of chelated peptide in serum, receptor binding affinity, internalization into the tumour cells, and dissociation from the tumour cells, should be performed before being translated into an in vivo evaluation. After the aforementioned assessments have been successfully completed, the radiolabelled peptides can be used to explore preclinical aspects, including those related to diagnosis and treatment. Along with these steps, further research is also needed to determine how dosing factors, including administration time, route, and rate of dosage absorption, could affect treatment effectiveness. What changes in cells and microenvironments may be generated by different radiation quality and dose rates, and by what parameters the absorbed dosage or its biological efficacy might be altered, are all questions that will be explored in the years to come.

The development of radioisotopes with beta and gamma emissions is crucial to the advancement of theragnostics, and the installation of non-power research reactors and isotope accelerators is necessary to achieve this goal. Numerous radiopharmaceuticals are currently in use for the treatment of inoperable cancer, and the development of molecular target drugs, which are crucial for medicines, is constantly increasing, which boosts the likelihood of developing radiopharmaceuticals for efficient diagnosis and treatment [171].

As PRRT’s primary anti-cancer mechanism of action involves inducing DNA damage, it is crucial to comprehend how various radionuclides impact the DNA. Unfortunately, there is still a lack of information regarding the specific DNA damages that are generated by PRRT. A very important study was published back in 2014 in which Chandan Kumar and colleagues showed that equivalent dosages of β radiation and γ-rays have different cell-killing efficacies. This can be a stepping stone to further the research, where a favourable type of radionuclide can be chosen to obtain satisfactory outcomes [172].Despite the potential advantages of peptides, they also have limitations, such as a short vascular half-life due to enzymatic destruction. As disrupting normal protein folding is another approach that could be used to boost the radio sensitivity of PRRT, this could compromise the cell’s capacity to use proteins necessary for survival after PRRT. Therefore, there is an increasing need for the development of synthetically modified peptides in order to circumvent such obstacles. Many efforts, such as the inclusion of stable D-amino acids instead of the comparatively less stable L-amino acids, as well as the use of pseudopeptide bonds as an extension of the side chains of peptides, have been investigated for radiopharmaceutical applications [170]. In addition, to maintain receptor-binding affinity, the insertion of a spacer has been used between chelating moieties and the binding sequence.

The successful delivery of radionuclides to the relevant clinical practice has required an appropriate carrier platform, such as peptides or polymers. With the great advantage of chemistry and a deep understanding of cellular biology, scientists have investigated several delivery platforms, such as (i) antibodies and their fragments, (ii) organic and (iii) inorganic nanoparticles, and (iv) microspheres, as alternatives for peptides. Moreover, the scientific community has shown greater interest in the development of hybrid radionuclide carriers as compared to individual delivery systems. Studies have shown that the integration of a single carrier into a hybrid platform leads to an increase in the loading capacity of radionuclides and enhancement of the ability to reach specific targeting sites. The development of efficient radionuclides with additional functions is required for further clinical use in the future.

## Figures and Tables

**Table 1:** The utilized radionuclides and their properties.

Radionuclide	Half-Life	Therapeutic Decay	Daughter Nuclide	Clinical Application	Ref.
^32^P	14.26 days	β^−^	^32^S	Myeloproliferative neoplasms	[71]
^89^Sr	50.53 days	β^−^	^89^Y	Osseous (bony) metastases of bone cancer	[72]
^90^Y	64.10 h	β^−^	^90^Zr	Liver tumours, primary hepatocellular carcinoma (HCC), and unresectable primary colorectal cancer	[73,74,75]
^166^Ho	26.76 h	β^−^	^166^Er	NETs, colorectal cancer, small hepatocellular carcinoma (SCC)	[76,77,78]
^117m^Sn	13.60 days	Isomeric transition	^117^Sn	Bone metastases	[79]
^99m^Tc	6.00 h	Isomeric transition	^99^Tc	HER2-positive breast cancer	[80]
^177^Lu	6.6 days	β^−^	^177^Hf	Metastatic castration-resistant prostate cancer	[81]
^223^Ra	11.4 days	α	^219^Rn	Advanced prostate cancer with bone metastases	[82]
^227^Th	18.7 days	α	^223^Ra	Haematological cancers, including CD22-positive B cell cancers and CD33-positive leukaemia	[83]
^153^Sm	46.2 h	β^−^	^153^Eu	Metastatic bone disease	[84]
^211^At	7.2 h	α	^207^Bi	Bone microtumours	[85]
^188^Re	16.9 h	β^−^	^188^Os	Painful bone metastases Medullary carcinoma	[86,87]
^131^I	8.0 days	β^−^	^131^Xe	Thyroid cancer	[88]
^111^In	2.80 days	Electron capture	^111^Cd	Oesophageal cancer	[89]

## Data Availability

Not applicable.
